# Mini-Thoracotomy Access Reduces the Incidence of Postoperative Atrial Fibrillation after Aortic Valve Replacement

**DOI:** 10.1186/1749-8090-10-S1-A77

**Published:** 2015-12-16

**Authors:** Hansraj R Bookun, Andrew Cheng, Chin Siew Lee, David Lee, Xiao Bo Zhang, Shivanand Gangahanumaiah, Marco Moscarelli, Giacomo Bianchi, Pier A Farneti, Marco Solinas

**Affiliations:** 1 Department of Vascular Surgery, University Hospital Geelong, Geelong, Australia; 2 Department of Cardiothoracic Surgery, University Hospital Geelong, Geelong, Australia; 3 Cardiology Research Unit, University Hospital Geelong, Geelong, Australia; 4 Cardiothoracic Department, Fondazione Toscana G. Monasterio, Massa, Italy

## Background/Introduction

Aortic valve replacement (AVR) is the current gold standard treatment for aortic stenosis. In the last 15 years popularity for minimally invasive (MI) AVR has grown exponentially. The most common techniques for MI-AVR are a partial sternotomy (PS) or a mini-thoracotomy (MT). Although MI access is technically more challenging and results in longer cross clamp and cardiopulmonary bypass times, MI has resulted in superior outcomes. Many publications have reported reduced postoperative pain, surgical trauma, blood loss, transfusion requirements, ventilation times, hospital stay with also earlier functional recovery and better cosmesis. Reductions in the incidence of postoperative atrial fibrillation (POAF) have also been shown.

## Aims/Objectives

Compare the incidence of POAF between MT and PS techniques in MI-AVR.

## Method

From January 2001 to January 2015, 1417 AVR cases were performed. Demographic and perioperative data were collected prospectively. A total of 523 patients underwent a full sternotomy or had past history of atrial fibrillation and were excluded from analysis. POAF was identified on the ward or ICU by continuous cardiac monitoring.

Homogeneity of the sample was tested using multivariate regression and Kolmogorov-Smirnov tests, which did not identify any statistically significant confounding variables. Descriptive statistics were used to characterize samples with regards to demographic and perioperative variables.

## Results

A total of 894 (475 males) patients met the inclusion criteria (309 PS and 585 MT cases). Overall, mean age was 70.9 ± 11.2 years. When compared to PS, MT had a shorter cardiopulmonary bypass (111 ± 38 vs. 117 ± 35; p = 0.038) and cross clamp time (80 ± 26 vs. 76 ± 30; p = 0.011). Incidence POAF was significantly lower in MT at 27.8% (n = 148) compared with 37.6% (n = 88), p = 0.007.

## Discussion/Conclusion

MI-AVR via MT approach significantly reduces POAF compared with PS approach.

